# Role of the intracerebroventricular injection α- klotho on food intake in broiler chicken: a novel study

**DOI:** 10.1016/j.psj.2024.104166

**Published:** 2024-08-06

**Authors:** Tahereh Eslam-aghdam, Shahin Hassanpour, Morteza Zendehdel

**Affiliations:** ⁎ Faculty of Veterinary Medicine, Science and Research Branch, Islamic Azad University, Tehran, Iran; † Division of Physiology, Department of Basic Sciences, Faculty of Veterinary Medicine, Science and Research Branch, Islamic Azad University, Tehran, Iran; ‡ Department of Basic Sciences, Faculty of Veterinary Medicine, University of Tehran, 14155-6453, Tehran, Iran

**Keywords:** α-klotho, hypophagia, NPY, broiler chicken

## Abstract

This novel study investigated the effects of intracerebroventricular (**ICV**) injection α- klotho and its interaction with neuropeptide Y (**NPY**) receptors on food intake in broiler chicken. This study included 4 experiments with 4 groups in each with 11 replicates per group. Birds were feed deprived 3 h prior injection, following injection returned to their cage and food provided. In experiment 1, group 1 received ICV injection of the saline and groups 2 to 4 received ICV injection of the α-klotho (1, 2, and 4 µg), respectively. In experiment 2, chicken received ICV injection of the saline, B5063 (NPY_1_ receptor antagonist, 1.25 µg), α-klotho (4 µg) and co-injection of the B5063 + α-klotho. In experiments 3 and 4, SF22 (NPY_2_ receptor antagonist, 1.25 µg), and SML0891 (NPY_5_ receptor antagonist, 1.25 µg) were injected instead of the B5063. Then consumed food was measured at 30, 60, and 120 min post the injection. Based on results, ICV injection of the α-klotho (2 and 4 µg) significantly decreased food intake (*P* < 0.05). Co-injection of the B5063 + α-klotho significantly amplified hypophagic effect of the α-klotho (*P* < 0.05). α-klotho-induced hypophagia was not influenced by SF22 or SML0891. These results suggest that α-klotho-induced hypophagia is mediated via NPY_1_ receptors in broiler chicken.

## INTRODUCTION

The klotho gene was accidentally discovered in mice in late 90's. Based on *in vitro* study, 2 forms of the klotho protein, membranous and soluble are detected (Oh et al., 2019). α-klotho is synthesized predominantly in the distal renal tubule, in the brain, parathyroid and pituitary gland. Also, α-klotho is found in serum, cerebrospinal fluid and urine in humans and mice (Martín-Núñez et al., 2017). α-klotho regulates nitric oxide synthesis pathway and insulin / insulin-like growth factor-1 signal inhibition. α-klotho promotes lipid oxidation, protects pancreatic β-cells from oxidative damage, increases energy expenditure, and facilitates insulin release (Oh et al., 2019). α-klotho is responsible with food intake and energy expenditure. Overexpression of the α-klotho in ^db/db^ mice attenuates hyperglycemia and hyperphagia. Genetic deletion of a α-klotho gene expression, declines temperature in brown adipose tissue, and rise food intake relative to body weight (Rao et al., 2019).

Food intake controls by peripheral signals from gastrointestinal tract and also at the central nervous system level in the brain. Neurotransmitters play key role on food intake, appetite regulation and energy expenditure (Mazzoni et al., 2022). Although some aspects of the food intake and appetite regulation mechanism are similar between animals, there are some dissimilarities among then (Motaghi et al., 2021). NPY/ agouti-related peptide (**AgRP**)-expressing neurons are located within the arcuate nucleus (**ARC**) of the hypothalamus and play an important role in feeding behavior and energy expenditure (Rao et al., 2019). NPY has a total of 6 receptors, with NPY_1_ and NPY_5_ receptors being specifically linked to the regulation of feed intake. Nevertheless, NPY_2_ is a type of auto-receptor that impacts hunger in animals that are lacking food. The ICV injection of NPY_1_ and NPY_5_ receptor antagonists led to a reduction in feed intake correlating with the dosage used. In contrast, NPY_2_ receptor blocker, increased the amount of food consumed by broiler chickens (Yousefvand et al., 2019).

In is reported, ICV injection of the α-klotho (2.0 μg) suppressed food intake, improved glucose profiles, and leads to loos body weight in type 1 and 2 diabetes models of mouse (Landry et al., 2020). α-klotho is a negative regulator of the NPY/AgRP expressing neurons in the ARC (Landry et al., 2020). However, it is reported intraperitoneally injection of the α-klotho (0.02 mg/kg) reduced adiposity, and elevated energy expenditure, with no changes in food intake in mice (Rao et al., 2019). Despite peripheral α-klotho has roles regulation of the metabolism, it cannot cross the blood brain barrier due to high molecular weight (Nakajima et al., 2016).

In mammals, fibroblast growth factor 23 regulates phosphate homeostasis in kidney by binding α-Klotho, a coreceptor of fibroblast growth factor 23 (**FGF23**) and Wang et al., (2018) reported that FGF23 regulates phosphate homeostasis in kidney by binding α-Klotho" in chicken. However, FGF23 mRNA expression pattern in chicken was clearly different from that in mammals and dietary phosphorus regulated the expression of FGF23 in a tissue-specific way. Although there is a concerted effort among scientists and researchers to find functional role of the α-klotho on food intake regulation, limit information exists on the physiological activities of the α-klotho in appetite regulation. Based on differences on central food intake regulation among avian and mammalian, and importance of the understanding the mechanisms of food intake in sight of comparative physiology, there is no report for role of the α-klotho in avian. Thus, this study was done to determine role of the ICV injection α- klotho and its interaction with NPY receptors on food intake regulation in broiler chicken.

## MATERIAL AND METHODS

### Animals

One hundred seventy-six meat-type (Ross- 308) day old chicken obtained from a domestic hatchery. The chicks were kept in groups for 2 d, then placed in solitary confinement for 5 d. Birds had free access to water and starter diet. The study protocol was approved by the Animal Ethics Committee of the Science and Research Branch of Islamic Azad University, Tehran, Iran (IR.IAU.SRB.REC.1402.206; 2023-09-04).

### ICV Injection

ICV injection was done on d 5. Head of the conscious chick held by an acrylic device. A hole was made in the stencil and placed on the skull in the right ventricular area (Jonaidi et al., 2012). The needle of the Hamilton syringe was inserted 4 mm into the skull. Injections were done in volume of the 10 µL with no stress (Saito et al., 2005). At the end of each experiment, the chicks were sacrificed with an intraperitoneal overdose (50 mg/kg) of sodium thiopental (Rotexmedica, Germany; according to AVMA Guidelines for the Euthanasia of Animals ‘No: S5.2.1.1’, Acceptable Methods; noninhaled agents). The accuracy of the injection on the right ventricle determined by decapitation at the end of the study and confirmed by the existence of Evans Blue in the injected area (Furuse et al., 1999).

### Grouping and Food Intake Measurement

This study included 4 experiments with 4 groups in each with 11 repeats for each group. Before study, birds were off feed for 3 h (FD_3_) and following injection, returned to their cage. In experiment 1, group 1 received ICV injection of the saline and groups 2 to 4 received ICV injection of α-klotho (1, 2 and 4 µg), respectively. In experiment 2, chicken received ICV injection of the saline, B5063 (NPY_1_ receptor antagonist, 1.25 µg), α-klotho (4 µg) and co-injection of the B5063 + α-klotho. In experiment 3, chicken received ICV injection of the saline, SF22 (NPY_2_ receptor antagonist, 1.25 µg), α-klotho (4 µg) and co-injection of the SF22 + α-klotho. In experiment 4, injections were saline, SML0891 (NPY_5_ receptor antagonist, 1.25 µg), α-klotho (4 µg) and co-injection of the SML0891 + α-klotho. Then the consumed food was measured at 30, 60 and 120 min post the injection (Madadi et al., 2023).

### Statistical Analysis

Food intake was determined based on % of body weight and analyzed using the repeated measures analysis of variance (**ANOVA**) using SPSS 21.0 for Windows (SPSS, Inc., Chicago, IL) and presented as mean ± SEM. To compare the means Tukey-Kramer test was used (*P* < 0.05).

## RESULTS

**Figure 1:**
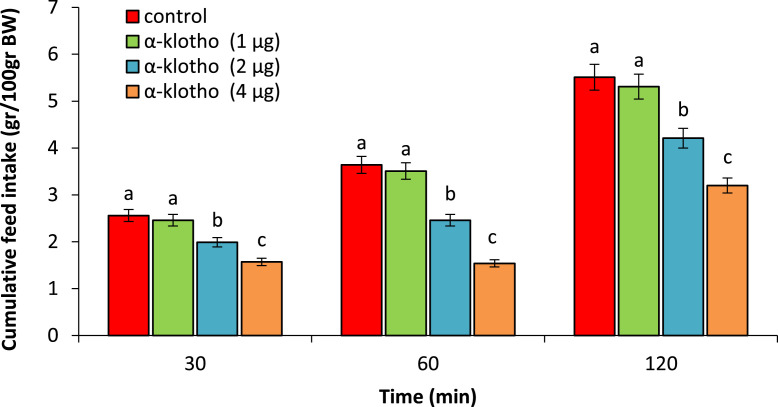
Effect of ICV injection of α-klotho (1, 2 and 4 µg) on cumulative food intake in neonatal chicken (n = 44). Data are expressed as mean ± SEM. Different letters (a–c) indicate significant differences between treatments (*P* < 0.05).

As seen in experiment 1, ICV injection of the α-klotho (1 µg) had no effect on food intake (*P* > 0.05) but hypophagia observed by ICV injection of the 2 and 4 µg comparison to control chicken (*P* < 0.05) (Figure 1).

**Figure 2:**
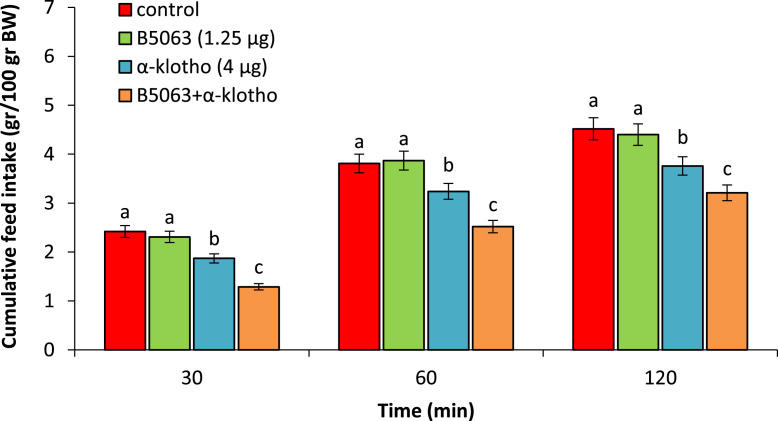
Effect of ICV injection of B5063 (1.25 µg), α-klotho (4 µg) and their combination on cumulative food intake in neonatal chicken (n = 44). BIBP-3226: NPY1 receptor antagonist. Data are expressed as mean ± SEM. Different letters (a, b and c) indicate significant differences between treatments (*P* < 0.05).

Based on experiment 2, hypophagia was observed following ICV injection of the α-klotho (4 µg) in comparison to control chicken (*P* < 0.05). Injection of the B5063 (1.25 µg) had no effect on cumulative food consumption (*P* > 0.05). Co-injection of the B5063 + α-klotho significantly amplified hypophagic effect of the α-klotho (*P* < 0.05) (Figure 2).

**Figure 3:**
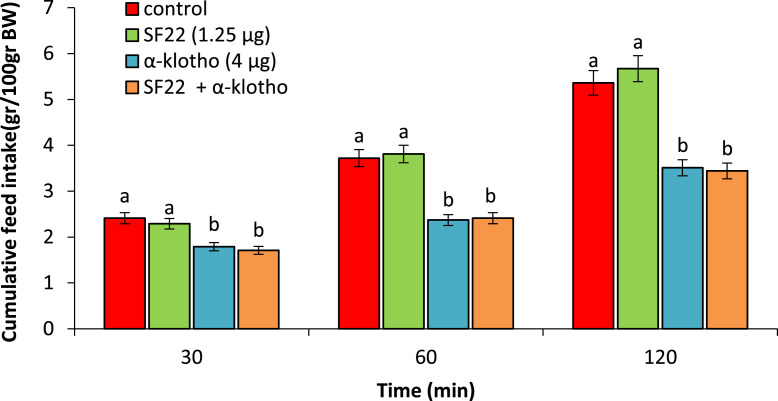
Effect of ICV injection of SF22 (1.25 µg), α-klotho (4 µg) and their cssombination on cumulative food intake in neonatal chicken (n = 44). BIIE 0246: NPY2 receptor antagonist. Data are expressed as mean ± SEM. Different letters (a and b) indicate significant differences between treatments (*P* < 0.05).

According to experiment 3, no significant change was seen following ICV injection of the SF22 (1.25 µg) on food intake compared to control chicken (*P* > 0.05). α-klotho (4 µg) significantly decreased food consumption compared to control group (*P* < 0.05). Co-injection SF22 + α-klotho had no effect on the α-klotho -induced hypophagia (*P* > 0.05) (Figure 3).

**Figure 4:**
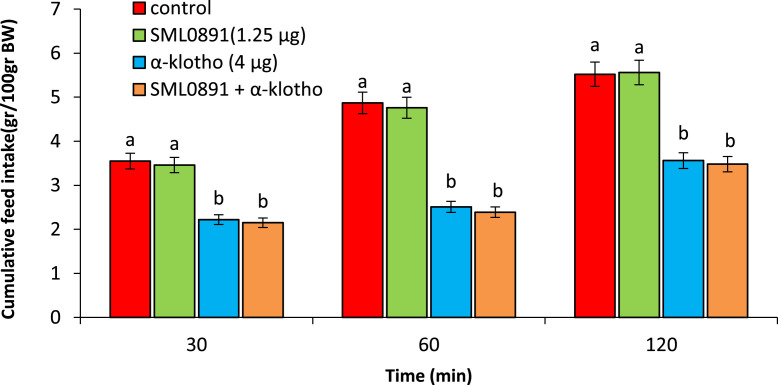
Effect of ICV injection of SML0891 (1.25 µg), α-klotho (4 µg) and their combination on cumulative food intake in neonatal chicken (n = 44). SML0891: NPY5 receptor antagonist. Data are expressed as mean ± SEM. Different letters (a and b) indicate significant differences between treatments (*P* < 0.05).

As seen in Figure 4, ICV injection of the SML0891 (1.25 µg) had no significant effect on food intake (*P* > 0.05).

Injection of the α-klotho (4 µg) significantly decreased food consumption comparison to control chicken (*P* < 0.05). Co-injection SML0891 + α-klotho had no significant effect on α-klotho-induced hypophagia (*P* > 0.05).

## DISCUSSION

This is the first report about the effects of effect of ICV of α-klotho and possible interactions with NPY receptors on appetite regulation in birds. Based on findings, ICV injection of α-klotho (2 and 4 µg) decreased food intake. Co-injection of the NPY_1_ receptor antagonsit + α-klotho amplified hypophagic effect of the α-klotho. α-klotho-induced hypophagia was not influenced by NPY_2_ or NPY_5_ receptors. Due to α-klotho's inability to cross the blood-brain barrier, central and peripheral α-klotho have independent physiological roles. Peripheral α-klotho improving adiposity in diabetic mice but central α-klotho has more important role in metabolic regulation. As first time, Landry et al., (2020) reported ICV injection of the α-klotho decreased food intake and body weight in diabetic mice which our finding was in agreement to this report.

Based on our finding, α-klotho-induced hypophagia is mediated via NPY_1_ receptors in broiler chicken and NPY_2_ and NPY_5_ receptors have no role on its hypophagic role. α-klotho is an antagonist of NPY/AgRP neurons. ICV injection of the α-klotho showed similar effects to NPY/AgRP inhibition (Meister et al., 2021). Despite NPY has 6 receptors, only the NPY_1_ and NPY_5_ receptors are involved in feed intake regulation. NPY_2_ is an auto receptor that influences appetite in food-deprived animals. ICV injection of the NPY_1_ and NPY_5_ receptor antagonists decreased feeding behavior in broiler chicken (Yousefvand et al., 2019). α-klotho involved in downregulating NPY/AgRP gene transcription and activity (Li et al., 2019). It is reported all POMC neurons in the hypothalamus responded to α-klotho. PI_3_kinase signaling is critical to α-klotho-mediated regulation of both NPY/AgRP and POMC neurons. α-klotho concentrations in the CSF decreased in neurological disorders which exhibit its role in energy expenditure and appetite regulation. ICV injection of the α-klotho improves hepatic lipid accumulation and central α-klotho inhibition rapidly impairs glucose clearance (Landry et al., 2020). It is also possible α-klotho regulates POMC neurons indirectly via presynaptic inputs. α-klotho direct inhibitory role in NPY/AgRP neurons may indirectly stimulate POMC activity via relieved NPY/AgRP-mediated inputs. Interestingly, NPY/AgRP→POMC connectivity is complex, highlighted by differences in the effects of spontaneous vs. stimulated NPY/AgRP neuron activity (Chen et al., 2018). Despite there are differences on central food intake regulation between avian and mammalian, it seems mediatory role of the α-klotho on food intake in avian is similar to mammalian.

## CONCLUSION

In conclusion, these findings are important form case of comparative physiology, in terms of the mechanisms of appetite regulation. These results suggested α-klotho-induced hypophagia mediates via NPY_1_ receptors in broiler chicken which was similar with reports in mammalian.

## DISCLOSURES

No potential conflict of interest was reported by the authors.
